# Targeted next-generation sequencing of sputum for diagnosis of drug-resistant TB: results of a national survey in Democratic Republic of the Congo

**DOI:** 10.1038/s41598-020-67479-4

**Published:** 2020-07-01

**Authors:** Michel Kaswa Kayomo, Vital Nkake Mbula, Muriel Aloni, Emmanuel André, Leen Rigouts, Fairouz Boutachkourt, Bouke C. de Jong, Nicolas M. Nkiere, Anna S. Dean

**Affiliations:** 1 National Tuberculosis Program et Laboratoire National de Référence des Mycobactéries (LNRM), Kinshasa, Democratic Republic of the Congo; 2 Department of Microbiology, Immunology and Transplantation, KU Leuven, Leuven, Belgium; 3 Institute of Tropical Medicine, Antwerp, Belgium; 4 Pôle de Microbiologie Médicale, Institut de Recherche Expérimental Et Clinique, UC Louvain, Brussels, Belgium; 5 World Health Organization, Kinshasa, Democratic Republic of the Congo; 6 Global TB Programme, World Health Organization, Geneva, Switzerland; 7 Faculté de Médecine, Université de Kinshasa, Kinshasa, Democratic Republic of the Congo; 8 Antwerp University, Antwerp, Belgium

**Keywords:** Antimicrobial resistance, Infectious-disease diagnostics, Tuberculosis

## Abstract

The surveillance of drug resistance among tuberculosis (TB) patients is central to preventing the spread of antimicrobial resistance. The Democratic Republic of the Congo (DR Congo) is classified by the World Health Organization (WHO) as a country with a high burden of TB and multidrug-resistant TB (MDR-TB), but there are no nationally representative data on drug resistance. In 2016–2017, a national survey of TB patients was conducted in 108 microscopy centres across all 11 provinces of the country using innovative molecular approaches. Sputum samples were collected from 1,545 new and 163 previously treated patients. These were tested by the Xpert MTB/RIF assay, followed by targeted next-generation sequencing performed directly on sputum. The prevalence of rifampicin resistance was low, at 1.8% (95% CI: 1.0–3.2) among new and 17.3% (95% CI: 11.9–24.4) among previously treated patients. Resistance to pyrazinamide, fluoroquinolones and second-line injectables was also low. The prevalence of resistance to isoniazid among rifampicin-susceptible patients was higher, at 6.6% (95% CI: 4.4–9.8) among new and 8.7% (95% : 3.2–21.2) among previously treated patients. Diagnosing and treating isoniazid-resistant patients remains a challenge, given that many will be missed by the current national diagnostic algorithm that is driven by detecting rifampicin resistance by Xpert MTB/RIF. This is the first nationwide survey incorporating targeted sequencing directly on sputum. It serves as a proof-of-concept for other settings that do yet have rapid specimen transport networks or capacity to conduct culture.

## Background

The surveillance of drug resistance among tuberculosis (TB) patients is central to combatting the global TB epidemic and preventing the spread of antimicrobial resistance. Data generated from surveillance can be used to guide the planning of TB diagnostic and treatment services, design appropriate treatment regimens, and monitor the effectiveness of control interventions. The End TB Strategy of the World Health Organization (WHO) calls for universal drug susceptibility testing. In settings where capacity for routine continuous surveillance is not yet sufficient, periodic surveys of a nationally representative sample of TB patients are recommended^1^.

The Democratic Republic of the Congo (DR Congo) is among the 30 high TB burden countries and 30 high multidrug-resistant (MDR) TB burden countries defined by WHO for the period of 2016–2020. Only four of these countries (Angola, Congo, DR Congo and Liberia) do not have any recent, high quality data on the burden of drug-resistant TB. WHO estimated that 6,000 new cases of RR-TB occurred in 2018, although only 787 were detected and reported^2^.

Molecular technologies overcome many of the challenges associated with conventional culture-based phenotypic drug susceptibility testing. The Xpert MTB/RIF assay (Cepheid, CA, USA) is a cartridge-based test that can be implemented at the peripheral level to rapidly diagnose *Mycobacterium tuberculosis* complex from sputum as well as detect rifampicin resistance. DR Congo was an early implementer of the GeneXpert platform, rolling out the Xpert MTB/RIF assay from 2011^3^. National drug resistance surveys have been successfully conducted in resource-limited settings based on Xpert MTB/RIF testing^4–6^. Next-generation sequencing is a reliable and efficient tool for the surveillance of resistance to both first-line and second-line drugs as well as providing information about the phylogenic characteristics of circulating strains, and is being increasingly incorporated into national drug resistance surveys^7–9^. In this article, we present the findings of the first national anti-TB drug resistance survey conducted in DR Congo in 2016–2017. This is the first national survey based entirely on targeted next-generation sequencing performed directly on sputum, without the need for culture.

## Methods

### Study design

This cross-sectional survey targeted new and previously treated pulmonary TB patients with sputum smear-positive pulmonary TB diagnosed in the country. The design of the survey was cluster-based, with the clustering unit defined by pre-existing health sanitary zones. Each health sanitary zone contained at least one diagnostic centre equipped with light microscopy for sputum smear examination. In view of logistics and available resources, the number of clusters to participate in the survey was set at 30. To select these clusters, a probability-proportional-to-size sampling was conducted using a list of the 515 health sanitary zones that existed in the country in 2012, covering 1,746 diagnostic centres. One additional cluster was purposively selected so that all 11 provinces that existed in 2012 were included. The 31 clusters comprised 108 microscopy centres.

The target sample size for new patients was 1,488, equating to 48 per cluster. This calculation was based on an anticipated prevalence of MDR-TB among new TB cases of 1.8%, a desired absolute precision of 1.25% at the 95% confidence interval, a design effect of 2 due to clustering, and expected losses of 15% due to reasons such as insufficient or contaminated samples. There was no separate sample size calculation for previously treated patients, who were recruited opportunistically until the sample size for new patients was reached. Enrolment of patients began in June 2016 and concluded in December 2017.

All newly registered sputum smear-positive new and previously treated pulmonary TB patients presenting to the survey sites, and from whom informed written consent was obtained, or from a legal guardian on their behalf, were eligible for inclusion in the survey. Cases of extra-pulmonary TB without pulmonary involvement were excluded. A questionnaire was administered by a trained interviewer at the time of enrolment, which included questions relating to demographics, TB treatment history (new or previously treated), social factors (such as living in an urban/rural setting, previous incarceration), and other health-related indicators (such as a history of smoking and HIV status). Ethical clearance for the survey was obtained from the National Ethics Committee of DR Congo and all research was performed in accordance with national guidelines for the diagnosis and treatment of TB.

### Laboratory methods

Sputum smear microscopy was performed after Ziehl–Neelsen staining, in accordance with national guidelines for routine diagnosis of TB. From all eligible patients diagnosed as sputum smear-positive, two additional sputum samples were collected. Spot and morning samples were preserved in 5 mL of Cetyl Pyridinium Chloride (CPC), while an additional spot sample was preserved in 96% ethanol at ratio of 1:1^10^.

All samples were stored at room temperature and transported in triple packaging by road or air to the nearest testing site for Xpert MTB/RIF, which was either a provincial-level laboratory or the National TB Reference Laboratory (NRL) in Kinshasa. The spot sample preserved in CPC was tested by Xpert MTB/RIF for identification of the *M. tuberculosis* complex and determination of rifampicin susceptibility status. For patients that were positive for *M. tuberculosis* complex, the ethanol-preserved sample was shipped by air from the NRL to the Institute of Tropical Medicine (ITM) in Antwerp, Belgium.

At the ITM, 200 µL of the ethanol-preserved samples were digested with proteinase K and DNA was extracted using the semi-automated Maxwell 16 FFPE DNA purification kit with a Maxwell machine (model 4.9, Promega), and eluted in 50 µL Maxwell elution buffer^11^. The DNA was sequenced using the Deeplex-MycTB kit (Genoscreen, Lille, France) according to manufacturer's instructions. This assay employs ultra-deep sequencing of a single, 24-plexed amplicon mix to identify the mycobacterial species, type strains of the *M. tuberculosis* complex, and detect mutations in 18 gene regions associated with resistance to first- and second-line drugs. Amplicons were purified with Agencourt AMPure XP magnetic beads (Beckman Coulter, Indianapolis, IN, USA) and quantified by Qubit dsDNA BR assay (Life Technologies, Paisley, UK). Paired-end libraries of 150-bp read length were prepared with the Nextera XT DNA Sample Preparation kit (Illumina Inc, San Diego, CA, USA) and sequenced on an Illumina MiSeq platform using standard procedures. Drug susceptibility status was extrapolated from the sequences using a cloud-based analytical platform provided by GenoScreen (version 7.7.9)^12^ which is informed by published reference datasets of genetic variants associated with drug resistance^13–16^, with priority given to the collaborative, curative database of ReSeqTB^17^. Sequencing data are available on the Sequence Read Archive of the National Center for Biotechnology Information as recalibrated BAM files (BioProject number PRJNA560875).

In addition, for patients identified as RR-TB by Xpert MTB/RIF, line probe assay was performed using Genotype MTBDR*plus* (Hain LifeSciences, Germany) as well as Sanger sequencing of the entire *rpoB* gene on the same DNA extract used for Deeplex-MycTB sequencing, as per the manufacturer’s instructions or standard ITM protocol^18^.

### Statistical analysis

Survey data were entered into a pre-designed Access database software and analysed using Stata Statistical Software: Release 14 (StataCorp, 2015, College Station, TX, USA). Rifampicin susceptibility status was classified according to sequencing results or, when these were not available, Xpert MTB/RIF results were used. For all other drugs, only sequencing data were used. The estimated prevalence of resistance and 95% confidence intervals were calculated by logistic regression using the “svy” command, taking into account the clustered design and incorporating sampling weights (target cluster size divided by the actual number enrolled in cluster) to account for under- and over-enrolment.

The sensitivity and specificity of Xpert MTB/RIF for detection of rifampicin resistance was calculated against sequencing as the gold standard. For samples with a rifampicin result available from both Xpert MTB/RIF and sequencing, the level of interrater agreement was assessed by the Kappa statistic.

Potential risk factors for rifampicin resistance were investigated through univariate logistic regression to calculate odds ratios and associated 95% confidence intervals and p values.

## Results

### Patient inclusion

**Fig. 1:**
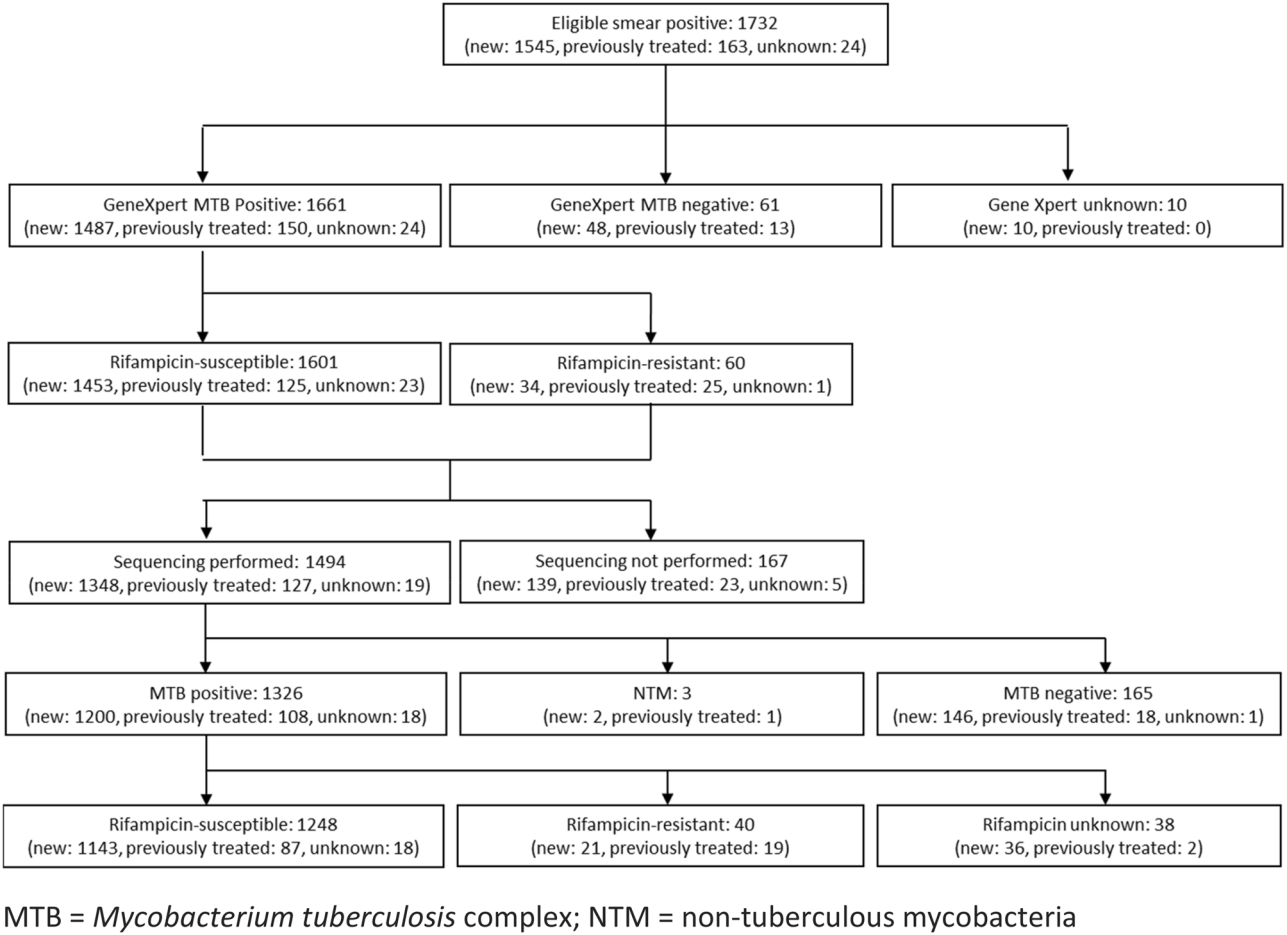
Flowchart of enrolled patients.

From June 2016 until December 2017, 1,732 sputum smear-positive pulmonary TB patients were enrolled, including 1,545 new patients, 163 previously treated patients and 24 patients with an unknown treatment status. Among these, 1,661 (95.9%) were positive for *M. tuberculosis* complex by Xpert MTB/RIF (Fig. 1).

The median age of patients that were positive for *M. tuberculosis* complex by Xpert MTB/RIF was 35 years (range 2–93 years) and more than half (59.7%) were male (Table 1). Around half of the patients lived in rural areas (52.4%) and 5.1% were HIV-positive, although HIV testing results were unavailable for 28% of patients.

**Table 1:** Characteristics of eligible patients (positive for *M. tuberculosis* complex by Xpert MTB/RIF).

	Number	Proportion (%)
**History of treatment**		
New	1487	89.5
Previously treated	150	9.0
Unknown	24	1.4
**Age**		
0–14 years	46	2.8
15–24 years	342	20.6
25–34 years	394	23.7
35–44 years	355	21.4
45–54 years	251	15.1
55–64 years	145	8.7
≥ 65 years	91	5.5
Unknown	37	2.2
**Sex**		
Male	991	59.7
Female	641	38.6
Unknown	29	1.8
**HIV**		
Negative	1112	67.0
Positive	85	5.1
Unknown	464	27.9
**Diabetes **		
No	1587	95.7
Yes	20	1.2
Unknown	51	3.1
**Setting **		
Urban	790	47.6
Rural	871	52.4
**History of imprisonment**		
No	1441	86.8
Yes	167	10.1
Unknown	53	3.2

Among the 1,661 patients with a positive result for *M. tuberculosis* complex by Xpert MTB/RIF, 1,326 were positive for *M. tuberculosis* by Deeplex-MycTB sequencing. A spoligotype profile was available for all of these, of which 1,160 could be assigned an international spoligotype (SIT) number. In addition, a single-nucleotide-polymorphism-based analysis included in the MycTB assay assigned a lineage for 738 samples. Lineages 4.3, 4.6.1, 3, 5 (*M. africanum*) and 1 accounted for respectively 429 (58%), 215 (29%), 52 (7%), 33 (4.5%) and 9 (1.2%) samples. This lineage distribution is in line with global data ^19^. Seventy cases of mixed *M. tuberculosis* infection were detected as well as three cases of non-tuberculosis mycobacteria (*M. avium and M. chitae*).

### Drug resistance patterns

Of the 1,494 sequenced samples, 403 (27%) had an incomplete drug susceptibility assessment across all target regions. This was probably due to a low bacterial load in these samples, as the mean coverage depth for this group of samples was 151 reads, compared to a mean coverage depth of 1,349 for all 1,494 samples. Sequencing runs typically had > 80% bases with a quality higher than Q30.

A result for rifampicin was available for 1,661 patients (1,487 new patients, 150 previously treated patients, and 24 patients of unknown treatment history) by Xpert MTB/RIF (Fig. 1). Among these 1,661 patients, sequencing results for rifampicin were also available for 1,288 patients (1,164 new patients, 106 previously treated patients, and 18 patients with unknown treatment history). The level of agreement for rifampicin testing between Xpert MTB/RIF and sequencing was 98% (kappa statistic = 0.79).The prevalence of resistance to rifampicin was 1.8% (95% CI: 1.0–3.2) among new patients and 17.3% (95% CI: 11.9–24.4) among previously treated patients (Table 2). Sequencing results for rifampicin were not available for 370 of the 1,661 patients with a positive result for *M. tuberculosis* complex by Xpert MTB/RIF (22.3%), mainly due to sample transport problems (167 patients) or very low bacterial load (203 patients) (Fig. 1). In 38 of the 40 samples with mutations (95%), the *rpoB* mutations were seen as a ‘fixed’ mutations, i.e. seen in more than 98% of the reads. The most frequent *rpoB* mutation was Ser450Leu (28/40, 70%) followed by mutations at codon 445 (Asn/Asp/Leu combined; 7/40, 17.5%). No mutations outside the *rpoB* region covered by Xpert MTB/RIF were identified. No association was found between rifampicin resistance and age, sex, urban versus rural setting, HIV status, diabetes or a history of imprisonment. Patients with a history of previous treatment for tuberculosis had an odds of rifampicin resistance that was 11.7 times higher (95% CI: 4.7–29.1) than new patients.

**Table 2:** Prevalence of resistance to first- and second-line drugs among TB patients.

	New patients		Previously treated patients		Rifampicin-susceptible patients		Rifampicin-resistant patients	
	Number with results	Prevalence, % (95% CI)	Number with results	Prevalence, % (95% CI)	Number with results	Prevalence, % (95% CI)	Number with results	Prevalence, % (95% CI)
Rifampicin resistance	1485	1.8 (1.0–3.2)	149	17.3 (11.9–24.4)	–	–	–	–
Any isoniazid resistance	1158	7.2 (4.8–10.7)	102	23.0 (14.5–34.5)	1213	6.7 (4.6–9.9)	38	71.5 (43.3–89.2)
Isoniazid resistance without rifampicin resistance	1112	6.6 (4.4-9.8)	84	8.7 (3.2-21.2)	1213	6.7 (4.6–9.9)	–	–
Rifampicin and isoniazid resistance (MDR-TB) 1	1133	0.9 (0.4–1.8)	102	15.8 (9.0–26.3)	–	–	37	73.7 (42.8–91.2)
Pyrazinamide resistance	1175	1.1 (0.6–2.1)	104	1.8 (0.5–6.7)	1217	0.3 (0.1–0.8)	38	21.6 (9.0–43.2)
Fluoroquinolone resistance	1042	0.1 (0–0.7)	93	0 (0–3.9)	1095	0 (0–0.7)	35	0 (0–10.0)
Injectable resistance	1128	0.3 (0.1–1.2)	100	0.8 (0.1–5.3)	1176	0.3 (0.1–1.2)	38	0 (0–9.3)
Fluoroquinolone or injectable resistance	983	0.4 (0.1–1.4)	88	1.0 (0.1–5.9)	1034	0.5 (0.1–1.4)	35	0 (0–10.0)
MDR-TB and fluoroquinolone and injectable resistance (XDR-TB)	983	0	88	0 (0–4.1)	–	–	35	0 (0–10.0)

The prevalence of resistance to isoniazid was higher that rifampicin resistance, including among rifampicin-susceptible patients where the prevalence was 6.7% (95% CI: 4.6–9.9). Isoniazid resistance was due to a mutation in the *inhA* gene (1/112, 0.9%), the promoter region of the *inhA* gene (26/112, 23.2%) or the *katG* gene (86/112, 76.8%). One case of combined mutations in the *inhA*-promoter and *katG* genes was detected. The prevalence of resistance to fluoroquinolones and second-line injectables was less than 1% among both rifampicin-susceptible and rifampicin-resistant patients. The prevalence of resistance to pyrazinamide was significantly higher in rifampicin-resistant patients (21.6%, 95% CI: 9.0–43.2, p < 0.001) compared to rifampicin-susceptible patients.

Among the 1,288 patients for which results for rifampicin were available by both Xpert MTB/RIF and MycTB deep sequencing (Table 3), the specificity of Xpert MTB/RIF compared to sequencing was high (99.5%) while sensitivity was lower (61.8%). Ten of eleven patients that were initially diagnosed as RR-TB by Xpert MTB/RIF but found to be rifampicin-susceptible by Deeplex-MycTB sequencing were also wildtype by MTBDR*plus* line probe assay and Sanger sequencing on the same DNA extract. Data on the bacillary load for these samples could not be retrieved from Xpert MTB/RIF data, but their sputum smear microscopy grades ranged from 1 + to 3 + . The six samples that were rifampicin-susceptible by Xpert MTB/RIF but RR-TB by Deeplex-MycTB all carried the most widespread mutation conferring rifampicin resistance, Ser450Leu, which can be reliably detected by Xpert MTB/RIF^20^.

**Table 3:** Rifampicin testing results by Xpert MTB/RIF and sequencing.

Xpert MTB/RIF	Sequencing		
	970280434594000Rifampicin-susceptible	Rifampicin-resistant	Total
970280434594000Rifampicin-susceptible	1237	6	1243
Rifampicin-resistant	11	34	45
Total	1248	40	1288

## Discussion

This first nationwide anti-TB drug resistance survey in DR Congo detected a low prevalence of RR-TB, at 1.8% (95% CI: 1.0–3.2) among new patients and 17.3% (95% CI: 11.9–24.4) among previously treated TB patients. This is in line with findings from four neighbouring countries with national drug resistance data available. In Rwanda, Tanzania, Uganda, Zambia, rifampicin resistance among new cases ranged from 0.9–2.8%^2^.

The prevalence of resistance to isoniazid among rifampicin-susceptible patients is more concerning, at 6.6% (95% CI: 4.4–9.8) among new patients and 8.7% (95% CI: 3.2–21.2) among previously treated patients. As in many countries, the current laboratory algorithm in DR Congo is based on screening by Xpert MTB/RIF to identify patients requiring further drug susceptibility testing and for whom an RR-TB treatment regimen is indicated. While this approach is justified, it means that patients who are eligible for the modified treatment regimen recommended by WHO in 2018 for isoniazid-resistant, rifampicin-susceptible TB will be missed^7^. These patients may experience poorer treatment outcomes and risk developing further resistance to other drugs^21^. The development of a simple, rapid, accurate and affordable test to detect isoniazid resistance at the peripheral level is of paramount importance. However, the level of isoniazid resistance (low- versus high-level) and its clinical relevance depends on the type of mutation(s) present. Lower minimal inhibitory concentrations (MICs) are seen for strains harbouring an *inhA*-promoter mutation alone, moderate MICs for *katG* mutation alone, and higher MICs for a combination of *inhA*-promoter and *katG* mutations^22,23^. Additional genes may also impact on the level of isoniazid resistance. When only low MICs are detected, isoniazid might still be effective when included as part of an appropriate treatment regimen^24,25^. In this survey, most cases of isoniazid resistance (76.8%) were due to *katG* mutations.

With the prevalence of resistance to fluoroquinolones being low, the recommended second-line regimens for RR-TB and isoniazid-resistant TB are likely to be effective in DR Congo. Although pyrazinamide resistance was higher among RR-TB patients, the prevalence was lower than observed in drug resistance surveys conducted in other countries^8,26^.

This is the first national survey to rely entirely on next-generation sequencing directly from sputum, providing resistance profiles for a range of drugs while bypassing the need for culture. Reliable results were obtained for the majority of the eligible patient population. Results were unavailable for 22.3% of eligible patients due to either sample transport problems or a very low bacterial load preventing successful sequencing. Given that rapid sputum transport networks and cold chains are not required, this approach should be considered for other resource-limited settings. Similar rates of losses have occurred in national surveys based on culture followed by whole genome sequencing^8^.

In a setting with a low prevalence of rifampicin resistance, the positive predictive value of Xpert MTB/RIF for rifampicin resistance is reduced^27^. In this survey, nearly one quarter (11 of 45, 24.4%) of cases detected to be RR-TB by Xpert MTB/RIF, and for which sequencing data were available, were rifampicin-susceptible by sequencing. This may reflect false rifampicin resistance by the Xpert MTB/RIF assay, mixed *M. tuberculosis* infection or clerical errors. We did not identify any silent *rpoB* mutations (mutations not conferring amino acid changes) that can lead to false detection of RR-TB by Xpert MTB/RIF. Low bacillary load at the time of Xpert MTB/RIF testing is also known to increase the risk of false RR-TB detection^28,29^, but these data could not be retrieved. Mixed *M. tuberculosis* infection with different strains present in different sputum samples^30^ or clerical errors are therefore plausible, given that Xpert MTB/RIF testing and sequencing were performed on different specimens a mixture of *M. tuberculosis* strains was observed in 5.7% of samples. Whenever an RR-TB patient belongs to a group at low risk of resistance, such as a new TB cases with no known contact with a patient with RR-TB, a second testing method should be used for confirmation and/or retesting with Xpert MTB/RIF to rule-out any clerical errors.

For the six cases that were rifampicin-susceptible by Xpert MTB/RIF but rifampicin-resistant by sequencing, possible reasons could be mixed infection in the sample tested by Xpert MTB/RIF with the proportion of mutant bacilli below the 65% detection limit of Xpert MTB/RIF^31,32^ or clerical errors.

This survey has several limitations. Firstly, it is restricted to sputum smear-positive pulmonary TB patients only. However, there is no evidence that the prevalence of drug resistance differs between smear-positive and smear-negative patients^33^. Secondly, discordant results between Xpert MTB/RIF and sequencing could not be further investigated with repeated testing. However, estimates of the prevalence of resistance was similar when calculated using Xpert MTB/RIF data or sequencing data.

## Conclusion

The prevalence of resistance to rifampicin, fluoroquinolones, second-line injectables and pyrazinamide among TB patients in DR Congo is encouragingly low. Diagnosing and treating isoniazid-resistant patients remains a challenge, given that many are susceptible to rifampicin and missed by the current national diagnostic algorithm that is driven by detection of rifampicin resistance by Xpert MTB/RIF. This is the first nationwide survey based on next-generation sequencing performed directly on sputum. This innovative molecular approach serves as a proof-of-concept for other settings that do yet have rapid specimen transport networks or capacity to conduct culture.
